# Assessment of Spontaneous Retinal Arterial Pulsations in Acute Central Retinal Vein Occlusions

**DOI:** 10.1155/2020/3107472

**Published:** 2020-06-09

**Authors:** Nicolas Arej, Vivien Vasseur, Elyse Jabbour, Anthony Manassero, Céline Giraud, Sébastien Bruneau, Yannick Le Mer, Martine Mauget-Faÿsse

**Affiliations:** ^1^Department of Ophthalmology (Vitreoretinal division), Rothschild Foundation Hospital, Paris 75019, France; ^2^Clinical Research Department, Rothschild Foundation Hospital, Paris 75019, France

## Abstract

Central retinal vein occlusion (CRVO) is a common retinal disease. Recent works mentioned spontaneous retinal arterial pulsations (SRAPs) as a feature of some CRVOs. This is a retrospective study on patients presenting with CRVO who were followed up for at least 6 months. The objective was to identify SRAP in the acute phase of the disease and determine their relationship with patients' characteristics and visual prognosis. A 10-second infrared film centered on the optic disc was recorded within a month of the onset of symptoms, and SRAPs were detected in two-thirds of the cases. Patients with SRAP were significantly younger than those without SRAP. Mean central macular thickness was significantly higher in the absence of SRAP, which was translated into a more severe macular edema; however, this difference faded with time. BCVA tended to be higher in the presence of SRAP at the 6-month follow-up when adjusted to baseline. This study demonstrates that SRAPs are a frequent finding, easily detected by infrared fundus video recording, and associated with a younger age and lesser macular edema.

## 1. Introduction

Central retinal vein occlusion (CRVO) is the second most frequent retinal vascular disease after diabetic retinopathy, with an annual incidence of 2 to 3 per 10,000 [1, 2]. It induces circulatory slow-down and upstream blood stasis appearing in the form of retinal hemorrhages [3]. CRVOs were classically separated into two clinical forms: ischemic CRVO (also possibly associated with cotton-wool spots) and nonischemic CRVO [4], the former being regarded as the more severe due to VEGF-mediated neovascular complications [5, 6].

More recently, a new classification was suggested by Pierru et al. distinguishing 2 types of CRVO: type A characterized by an acute low blood flow and type B which settles more slowly [7]. Type A has been particularly associated with a younger age at presentation, the presence of a paracentral acute middle maculopathy (PAMM), a concomitant cilioretinal artery occlusion, and/or a pulsatile arterial filling. Type B is more likely to occur in older patients, usually with high blood pressure and multiple hemorrhages on fundus examination. Macular edema can be seen in both types; while it is regarded as the result of an altered blood-retina barrier in type B, it looks more like the extension of a more predominant papillary edema in type A. Hence, type A CRVOs are theoretically expected to have a more favorable evolution due to their acute onset except for extreme cases of macular infarction [8]. However, to the best of our knowledge, this hypothesis has not been yet evaluated in clinical studies.

The purpose of this study was to determine the frequency of spontaneous retinal arterial pulsations (SRAPs)—a feature of type A CRVO—in early examinations following CRVO and to assess the possible relationship between the presence of SRAP and the best-corrected visual acuity (BCVA) at presentation, as well as its progression over time.

## 2. Materials and Methods

This was a retrospective study that consisted of reviewing the records of patients who presented to the Rothschild Foundation Hospital (Paris, France) between 2012 and 2018 with a CRVO and were followed up at the same institution for at least 6 months.

Patients who presented within a month after the onset of symptoms and underwent a 10-second fundus video in infrared (IR) mode centered on the optic nerve head and central retinal vessels, using Spectralis HRA imaging (Heidelberg Engineering GmbH, Heidelberg, Germany), were included in this study. This fundus camera is able to acquire 8.8 images per second in real time [9]. The recordings were evaluated separately by two examiners (VV and MMF) to look for SRAP, and the patients were then grouped accordingly: “pulse +” and “pulse −.”

BCVA using the logMAR scale and the clinical signs of CRVO, i.e., the presence and extent of macular and papillary edema, the presence of PAMM and serous retinal detachment (SRD) on OCT B-scans were assessed at baseline and at 6 months. Exclusion criteria comprised CRVOs that were more than one month old at presentation, patients with media opacities preventing clear fundus imaging, and those with amblyopia or other conditions potentially associated with decreased visual acuity.

Statistical analyses were conducted using the SPSS version 22.0 (IBM Corp., Armonk, NY, USA). Descriptive statistics were reported as the mean and standard deviation for continuous variables. A chi-square test was used to compare frequencies and a *t*-test for means. A *p* value <0.05 was considered to be statistically significant. A simple linear regression model was applied to show trends in progression of visual acuity in both “pulse +” and “pulse −” groups after adjustment for baseline BCVA.

A local review board approval was obtained for this study, and all the investigations adhered to the tenets of the Declaration of Helsinki.

## 3. Results

Thirty-four eyes of 34 patients were included after meeting the inclusion criteria, in particular with regard to the availability of their data at baseline and at 6-month follow-up. Their characteristics are shown in Table 1. SRAP was detected in 23 (68%) eyes (see Video 1 and Video 2 in the Supplementary Material for examples of positive and negative SRAP, respectively).

Table 2 provides a comparison between patients of the “pulse +” and “pulse −” groups. Patients with SRAP appeared to be about 20 years younger than “pulse −” patients (*p*=0.001). However, there was no statistically significant difference regarding gender or associated conditions such as ocular hypertension, systemic hypertension, and diabetes mellitus. PAMM was not statistically more frequent in the “pulse +” group, and no cases of cilioretinal artery occlusion were observed.

**Figure 1:**
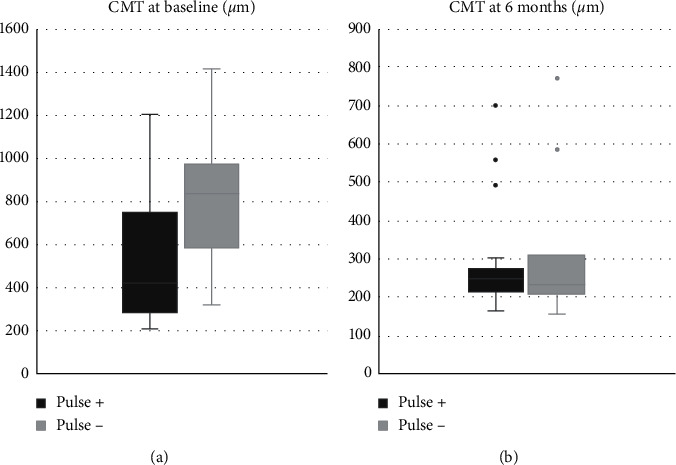
Mean central macular thickness (CMT) in *μ*m in the “pulse +” and “pulse −” groups: (a) at baseline and (b) at 6 months.

Both macular and papillary edemas were not significantly more frequent in the “pulse −” group at baseline or at 6 months. Nevertheless, the mean central macular thickness (CMT) was significantly higher in the “pulse −” group compared with the “pulse +” group at baseline (822.55 *μ*m vs 530.83 *μ*m, *p*=0.012) but not at 6 months, as shown in Figure 1. Also, SRD was numerically more frequent in the “pulse –” group at baseline (81.82% vs 43.48%, *p*=0.064), but this difference just failed to achieve statistical significance; there was no difference at 6 months.

**Figure 2:**
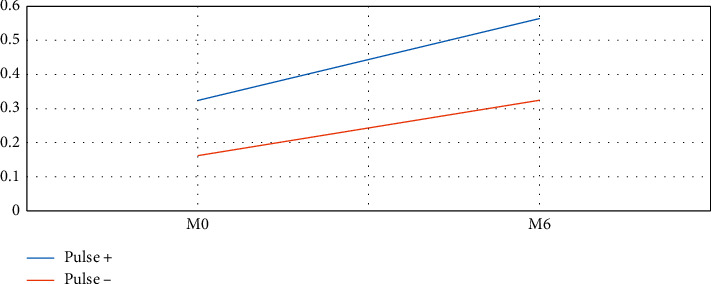
Evolution of the best-corrected visual acuity (BCVA) represented in decimal scale, between baseline (M0) and 6 months (M6), in patients of the “pulse +” and “pulse −” groups.

When macular edema developed and was associated with a drop of vision below 20/30, treatment was indicated. In pseudophakic eyes with no history of high intraocular pressure, intravitreal dexamethasone was injected and renewed if needed after at least 4 months, whereas in phakic eyes, 3 monthly intravitreal injections of anti-VEGF (ranibizumab or aflibercept) were performed and repeated according to a treat-and-extend regimen. No statistically significant difference was found between the 2 groups regarding the number of intravitreal anti-VEGF or dexamethasone implant injections used to manage macular edema. As for BCVA, it was slightly but not significantly higher in the “pulse +” group at baseline; the difference became wider and nearly significant at 6 months (*p*=0.075).  Figure 2 reveals a tendency towards a more important gain in visual acuity in patients with SRAP at 6 months, but this was not statistically significant (*p*=0.20) based on a linear regression model adjusted for baseline BCVA.

## 4. Discussion

CRVO is a common retinal vascular disease that may vary in presentation. Many theories have been provided to explain the observed clinical variety [10]. SRAP is a frequent finding in acute CRVO and was detected in more than half the cases in our study. It can be easily recorded using modern imaging techniques. Heidelberg Spectralis provides an accessible infrared video imaging capability that can acquire 10-second fundus films in high resolution. This option is particularly interesting for detecting SRAP. Pierru et al. have linked SRAP with type A CRVO in its early stage and described it “as if the arterial flow was hitting against the venous obstruction” and thus as the reflection of an acute low flow rate [8].

The findings of this study suggest that SRAP and, secondarily, type A CRVO occur more likely in younger adults and are associated with less pronounced macular edema. The current understanding of macular edema relates it to a disruption of the blood-retinal barrier [11]; this barrier seems, then, to be less altered in type A CRVO as initially suggested by Pierru et al. [7]. Nevertheless, the CMT of patients with SRAP becomes comparable to that of other patients over time. However, this cannot be attributed to the natural history of the disease since the patients in this study received intravitreal treatments when they developed macular edema.

Similarly, conclusions cannot be drawn regarding the progression of visual acuity in our study eyes, not only because no statistically significant difference was observed between the groups but also due to the presence of potential confounding factors, such as the compliance to treatment, the delay between intravitreal injections and assessment, and the development of cataract during the follow-up period. Although the latter condition is not likely to have a major effect over 6 months, it should necessarily be taken into consideration in a more extended study.

Another limitation of this study is its retrospective design. Despite this, we believe that it has provided some relevant results that would be helpful in establishing protocols for prospective studies involving more patients with longer follow-ups, in order to better understand the role of SRAP in different types of CRVO. In the event of larger investigations confirming our findings, SRAP would be considered as a prognostic biomarker of CRVO.

## 5. Conclusions

In conclusion, we observed that SRAP is a common and early distinctive sign of a CRVO category that occurs in relatively young patients with sufficient compensatory capacity that allows them to possibly have better and faster recovery. The hemodynamic mechanisms underlying this phenomenon remain presumptive; they may include acute low flow rate and relative preservation of the blood-retinal barrier. Therefore, further research is needed to establish a definitive relationship between SRAP and the prognosis of CRVO.

## Figures and Tables

**Table 1:** Patients' characteristics (*n* = 34).

Mean age (±SD)	60.5 ± 17.4
Gender	
Men	20 (58.82%)
Women	14 (41.18%)
Systemic hypertension	10 (29.41%)
Ocular hypertension/glaucoma	4 (11.76%)
Diabetes mellitus	2 (5.88%)
Category based on IR recording	
Pulse +	23 (67.65%)
Pulse −	11 (32.35%)

SD: standard deviation.

**Table 2:** Patients' characteristics, diagnostic features, and follow-up parameters compared between “pulse +” and “pulse −” groups.

	Pulse +	Pulse −	*p* value
Mean age in year ± SD	54.30 ± 16.60	73.45 ± 10.76	0.001^*∗*^
Male-to-female ratio	14 : 9	6 : 5	0.505
Systemic hypertension	21.74%	45.45%	0.232
Ocular hypertension/glaucoma	17.39%	0%	0.280
Diabetes mellitus	8.69%	0%	0.451
PAMM	52.17%	27.27%	0.271
Papillary edema at baseline	56.52%	63.63%	0.693
Papillary edema at month 6	0%	9.09%	0.483
Macular edema at baseline	60.87%	90.91%	0.113
Macular edema at month 6	13.04%	27.27%	0.363
Mean CMT at baseline (*μ*m) ± SD	530.83 ± 294.16	822.55 ± 304.62	0.012^*∗*^
Mean CMT at month 6 (*μ*m) ± SD	282.65 ± 127.29	305.73 ± 192.89	0.679
SRD at baseline	43.48%	81.82%	0.064
SRD at month 6	13.04%	18.18%	0.692
Mean number of anti-VEFG injections	3.5	2.8	0.349
Mean number of dexamethasone implants	1.33	1.50	0.725
Mean BCVA at baseline (logMAR)	0.80	1.10	0.211
Mean BCVA at month 6 (logMAR)	0.40	0.76	0.075

SD: standard deviation, PAMM: paracentral acute middle maculopathy, CMT: central macular thickness, SRD: serous retinal detachment, VEGF: vascular endothelial growth factor, and BCVA: best-corrected visual acuity; ^*∗*^*p* value <0.05.

## Data Availability

The data used to support the findings of this study are available from the corresponding author upon request.

## Supplementary Materials

Supplementary Materials Video 1.mp4 (10 seconds, 4.11 MB): central retinal vein occlusion with spontaneous retinal arterial pulsations. Video 2.mp4 (10 seconds, 3.53 MB): central retinal vein occlusion without spontaneous retinal arterial pulsations.
